# Evaluation of Blueberry Juice in Mouse Azoxymethane-Induced Aberrant Crypts and Oxidative Damage

**DOI:** 10.1155/2014/379890

**Published:** 2014-09-03

**Authors:** Isela Álvarez-González, Fernando Garcia-Melo, Verónica R. Vásquez-Garzón, Saúl Villa-Treviño, E. Osiris Madrigal-Santillán, José A. Morales-González, Jorge A. Mendoza-Pérez, Eduardo Madrigal-Bujaidar

**Affiliations:** ^1^Laboratorio de Genética, Escuela Nacional de Ciencias Biológicas, IPN, Unidad Profesional A. López Mateos, Avenida Wilfredo Massieu s/n, Zacatenco, Colonia, Lindavista, CP 07738, México, DF, Mexico; ^2^Departamento de Biología Celular, Centro de Investigación y Estudios Avanzados, IPN, Avenida Instituto Politécnico 2508, Colonia San Pedro Zacatenco, Del. Gustavo A. Madero, CP 06360, México, DF, Mexico; ^3^Laboratorio de Medicina de la Conservación, Escuela Superior de Medicina, IPN, Plan de San Luis y Díaz Mirón s/n, Casco de Santo Tomás, Del. Miguel Hidalgo, CP 11340, México, DF, Mexico; ^4^Laboratorio de Química Ambiental. Escuela Nacional de Ciencias Biológicas, IPN, Unidad Profesional A. López Mateos, Avenida Wilfredo Massieu s/n, Zacatenco, Colonia Lindavista, CP 07738, México, DF, Mexico

## Abstract

Blueberry is a plant with a number of nutritional and biomedical capabilities. In the present study we initially evaluated the capacity of its juice (BJ) to inhibit the number of aberrant crypts (AC) induced with azoxymethane (AOM) in mouse. BJ was administered daily by the oral route to three groups of animals during four weeks (1.6, 4.1, and 15.0 *μ*L/g), respectively, while AOM (10 mg/kg) was intraperitoneally injected to the mentioned groups, twice a week, in weeks two and three of the assay. We also included two control groups of mice, one administered distilled water and the other the high dose of BJ. A significant increase of AC was observed in the AOM treated animals, and a mean protection of 75.6% was determined with the two low doses of BJ tested; however, the high dose of the juice administered together with AOM increased the number of crypts more than four times the value observed in animals administered only AOM. Furthermore, we determined the antioxidant potential of BJ with an *ex vivo* DPPH assay and found a dose-dependent decrease with a mean of 19.5%. We also determined the DNA oxidation/antioxidation by identifying 8-hydroxy-2′-deoxyguanosine adducts and found a mean decrease of 44.3% with the BJ administration with respect to the level induced by AOM. Our results show a complex differential effect of BJ related to the tested doses, opening the need to further evaluate a number of factors so as to determine the possibility of a cocarcinogenic potential.

## 1. Introduction

Colon cancer refers to malignant tumors originated in the internal epithelial lining of the organ [1]. This disease has been associated with the accumulation of mutations including those in the K-ras, APC, and p53 genes as well as with deletions and gains in various chromosomes, in addition to epigenetic changes such as those related with hypo- and hypergene methylation [2]. In regard to histopathological features of the disease, one of the initial manifestations is the presence of aberrant crypts (AC), which may be found as a single abnormal crypt or as multiple crypts (ACF). These lesions can be observed along the colon of rodents and humans; they are characterized by a bigger size than the normal crypts, a thick layer of epithelial cells which often stain intensely, an oval lumen, and an enlarged pericryptal space [3]. One of the most used compounds for studying AC in experimental models is azoxymethane (AOM), an intermediary metabolite of dimethylhydrazine that gives rise to methyl diazonium and methyl carbonium, chemicals which are known to damage a number of biomolecules and may induce colon cancer [4].

During the carcinogenic process, oxidative stress is an event that may play a significant role, as shown by the induction of nitric oxide which in combination with the superoxide radical may generate peroxynitrite and hydroxyl molecules, which in turn may cause nitration, nitrosylation, and oxidation in proteins, DNA, and other biomolecules [5]. Also, in concordance with these observations, in a previous report we determined an increase in the level of lipid and protein oxidation which corresponded to the increase in the number of AC induced by AOM in mice [6].

Besides, diet, age, lifestyle, and other factors have been reported as risk factors for the development of the disease, suggesting then the potential relevance of applying chemopreventive measures related with the action of vegetables and fruits, because such strategy involves a variety of actions that can be exerted in different steps of the carcinogenic process [7]. In the case of colon carcinogenesis, the comprehension of pathogenic steps such as DNA damage, oxidative stress, chronic inflammation, and histological changes is relevant for the selection of early biomarkers that may show the efficacy of the studied chemopreventive agent.

In the present report we evaluated the effect of blueberry juice (BJ) (from* Vaccinium corymbosum *L.) on the damage induced by AOM in the colon of mouse. Blueberry is a perennial shrub 2 to 5 m high which possesses almost spherical, dark blue fruits 0.7 to 1.5 cm diameter which contain a number of vitamins and minerals as well as phytochemicals such as anthocyanins, flavonols, flavanols, stilbenes, phenolic acids, and proanthocyanidins [8].

The consumption of the studied plant has been increasing in natural or industrialized forms, including its use in bakery products, different types of syrups and alcoholic beverages, preserves, and coloring additives [9]. With respect to biomedical properties, several effects have been reported for different parts of* Vaccinium*, including antioxidant, antimicrobial, antiviral, anti-inflammatory, antihypertensive, and antiacetylcholinesterase activities [10, 11]. In regard to antigenotoxic and chemopreventive activities of* Vaccinium*, reports have shown an antiproliferative potential in prostate and colon cancer cellular lines, a protective effect of their enzymatic hydrolyzates against hydrogen peroxide-induced oxidative damage in Chinese hamster lung cell line, as well as* in vivo* reduction of benzo(a)pyrene induced genotoxic damage by ethanol extracts of the plant [11–13]. Moreover, studies with berry plants or with a number of their constituents have suggested a potential to inhibit carcinogenic damage [14, 15]. In this aspect, different molecular and cellular events induced by berry fruits have been proposed as mediators in the prevention of the carcinogenic process, including their antioxidant potential.

The above-mentioned information suggests the pertinence of extending studies in regard to the chemopreventive capacity of blueberry, particularly* in vivo*; therefore, the initial aim of the present report was to evaluate the capacity of BJ to inhibit the number of AOM-induced AC in the colon of mice and, secondly, to explore whether such an effect could have a relation with the BJ effect against oxidative damage. For this last purpose we determined its capacity to inhibit the percentage of the DNA adduct, 8-hydroxy-2′-deoxyguanosine (8-oxo-dG) induced by AOM, as well as its capacity to capture the 2,2-diphenil-1-picrilhydracil radical (DPPH) in mice.

## 2. Material and Methods

### 2.1. Chemicals and Animals

The following chemicals were purchased from Sigma-Aldrich Chemicals (St. Louis, MO, USA): AOM, PBS, proteinase K, RNAse, trichloroacetic acid (TCA), trizma base, albumin standard, thiobarbituric acid (TBA), triethylamine, hexane, potassium chloride, ethyl acetate, sodium acetate, vitamin E, quercetin, cyanidin-3-glucoside, Folin-Ciocalteu reagent, and methylene blue. Anti-8-oxo-dG (4354-MC-050) was purchased from Trevigen, Inc. (Gaithersburg, MD, USA). Formaldehyde, HCl, NaCl, sodium citrate, xylene, and ethanol were acquired from Fermont (Mexico City).

Blueberry was obtained from a pesticide-free cultivation in Zacatlán, Puebla, a town located 200 km north of Mexico City. The fruits were kept at −70°C, and, immediately before the assay, the juice was mechanically obtained by pressing the fruit with a mortar, followed by filtering it in gauze to eliminate gross material.

### 2.2. Phytochemical Identification

Several specific groups of phytochemicals have been identified as being mainly involved in the biomedical responses of blueberry; therefore, in the present report we made the identification of total phenolic compounds, flavonoids, and anthocyanins.

Initially, BJ was treated in a batch reactor with pectinase (Pectinex 3XL, 200 ppm) for 2 h at 55°C. The resulting product was passed through a 50 *μ*m filter and then it was lyophilized and powdered. Lyophilized BJ extracts were fractionated in a silica gel chromatographic column (embedded with hexane) and neutralized with triethylamine. Separations were made with gradients ranging from a mixture of hexane/ethyl acetate (1 : 1) to the use of ethyl acetate with increasing proportions of a mixture of methanol/water 1 : 1, until only the mixture methanol/water was used. Presently, fractions of BJ are in process to be analyzed with the methods of HPLC and ^1^H-NMR.

#### 2.2.1. Total Phenolic Content

The concentration of phenolics was determined by the method of Folin-Ciocalteu [16] with some modifications. The reaction mixture was prepared with 0.5 mL of BJ extract (0.2 g/mL), 2.5 mL of 10% Folin-Ciocalteu's reagent, and 2.5 mL of 7.5% sodium bicarbonate. The samples (in triplicate) were incubated at 45°C for 45 min and left at room temperature for 2 h in the dark before determining the absorbance at 750 nm. The same procedure was made with vitamin E to construct a calibration curve. Finally, the concentration of phenolics for each extract was extrapolated in the calibration curve and expressed as mg of vitamin E equivalents/100 g of extract.

#### 2.2.2. Total Flavonoid Content

For this determination we used the aluminum chloride colorimetric method previously reported [17] with some modifications. The tested samples were formed by 1 mL of a methanol BJ extract solution (1 mg/mL), plus 1 mL of 2% aluminum chloride. Samples (in triplicate) were left for an hour at room temperature before registering the absorbance at 510 nm. With a similar procedure we constructed a calibration curve for the standard solution (quercetin) where the readings of the extracts were extrapolated to finally express the flavonoid content as mg of quercetin equivalents/100 g of extract.

#### 2.2.3. Monomeric Anthocyanin Content

The anthocyanin pigment content was determined by the pH differential method [18], which is based on the reversible change of color with respect to the change in pH. The tested fractions were diluted with potassium chloride 0.025 M at pH 1 and with sodium acetate 0.4 M at pH 4.5; then, absorbance was determined at 520 and 700 nm after 30 min. Monomeric anthocyanins were calculated as mg of cyanidin-3-glucoside equivalents/100 g of BJ using the extinction coefficient of 26900 L mol^−1^cm^−1^ and a molecular weight of 449.2 g/mol. Cuvettes of 1 cm path length were used.

The obtained results were the following: total phenolic content = 350 ± 0.09 mg VE equivalents/100 g of BJ, flavonoid = 250 ± 1.0 mg of quercetin equivalents/100 g of BJ, and monomeric anthocyanins = 96.1 ± 0.01 mg of cyanidin-3-glucoside equivalents/100 g of BJ. These results show a significant amount of the evaluated phytochemicals and suggest that they may be involved in the determined antioxidant and chemopreventive results.

### 2.3. Experimental Protocol in Mouse

For the study we used 88 male mice (Swiss Webster) with a mean weight of 30 g. They were placed in metallic cages at 22 ± 2°C, 50–60% relative humidity, and in a 12 h dark-light cycle. Mice were permitted to freely consume food (Rodent Lab Chow 5001, Purina) and water.

The experiment was approved by the Committee of Ethics of the National School of Biological Sciences. Six groups with 8 mice each were used to evaluate the number of AC and the level of 8-oxo-dG. The tested doses of BJ (1.6, 4.1, and 15 *μ*L/g) correspond, respectively, to the consumption of 0.4, 1.2, and 3 glasses of 250 mL for a human of 65 kg. According to the selected doses of BJ and to the animal weight, we administered the corresponding volume of juice to each mouse by the intragastric route. One group was daily administered 0.3 mL of distilled water during the four weeks of the assay; another group was daily administered the high tested dose of BJ (15 *μ*L/g) during the whole assay; one more group was intraperitoneally (ip) injected with AOM (10 mg/kg) twice a week the second and third week of the assay; finally, three other groups were daily administered BJ with 1.6, 4.1, and 15 *μ*L/g each during the four weeks of the study as well as being ip injected 10 mg/kg of AOM, twice a week, in the second and in the third week. We had previously determined that the described experimental model is appropriate for both, to show the increase of AC by AOM and their inhibition by the tested chemopreventive agents [6].

The other 40 mice were used to determine the antioxidant capacity of BJ by applying the* ex vivo* DPPH assay. They were organized in five groups with 8 individuals each and orally administered as follows: one group 0.3 mL of distilled water, another group 3.1 *μ*L/g of vitamin E, and the last three groups BJ (1.6, 4.1, and 15 *μ*L/g). Blood was collected after 2 h of treatment by a retro-orbital puncture.

#### 2.3.1. Determination of AC

Mice were cervically dislocated at the end of the treatment; then, their colon was dissected and washed in PBS. The AC determination was made in the whole colon of each of the 8 animals per group. For this purpose we extended the organ in a Petri dish with solidified paraffin at the bottom and fixed it in 10% formaldehyde made in PBS for 24 h. After that, the organs were stained with 4% methylene blue (diluted in PBS) for 15 min, and the number and distribution of crypts were registered at 100x magnification.

#### 2.3.2. Immunohistochemical Determination of 8-Oxo-dG

For this purpose we followed the method described by Moreira et al. [19] with some modifications. Colon tissue sections, 3 *μ*m thick, were deparaffinized and hydrated gradually. Each sample was incubated with proteinase K (50 *μ*g/mL, 1 h at 37°C). The sections were then placed in a solution made with RNAse A 100 *μ*g/mL containing 150 mM NaCl and 15 mM sodium citrate at 37°C for 1 h. The tissue was washed twice with PBS, and, after DNA denaturation with HCl 2 N for 5 min, the process was neutralized with trizma base 1 M for 5 min and the tissue was washed twice in PBS. Nonspecific binding was blocked by incubating the slides for 60 min in 10% goat serum in PBS. Primary antibodies anti-8-oxo-dG diluted 1 : 250 were incubated overnight at 4°C followed by the secondary antibody Ig and the fluorescein-linked whole antibody at 37°C for 2 h. Tissue images were captured by an epifluorescent microscope (Axioskop 50 C. Zeiss) equipped with a filter (***γ*** = 365–395 nm) for detecting DAPI blue staining nuclei and another (***γ*** = 450–490 nm) for detecting the 8-oxo-dG adducts by the FICT green color. Fluorescent samples were analyzed in a Zeiss-510 laser scanning confocal microscope (Carl Zeiss, Inc., Jena, Germany) to confirm the coexistence of 8-oxo-dG in the nucleus. After confirming the colocalization patterns, we captured tissue images through an optical microscope (Olympus 1X70, Olympus Europe GmbH, Hamburg, Germany). Subsequently, tissue images were quantified in ten randomly selected fields (magnification 1000x) per individual sample. Adduct positive nuclei were quantified with respect to the total number of nuclei by using image analysis software (Analysis Soft Imaging System GmbH).

#### 2.3.3. DPPH Assay* Ex Vivo*

For this assay we followed the method of Chrzczarnowicz et al. [20] with some modifications. The blood sample from each mouse was centrifuged at 1500 ×g at 4°C for 10 min to obtain the serum. Then, 200 *μ*L of this was mixed with 200 *μ*L of acetonitrile 9.5 M and centrifuged at 9500 ×g at 4°C for 10 min. After this step, 25 *μ*L of the supernatant (deproteinized serum) was added to 5 *μ*L of the radical DPPH 0.01 M plus 970 *μ*L of methanol and left in the dark, at room temperature, for 30 min. Finally, absorbance was registered at 517 nm. In this assay the DPPH radical, which has an unpaired electron, was of a blue-violet color and tended to lose color turning to pale yellow.

#### 2.3.4. Statistical Analysis

Statistical significance of the obtained data was determined by applying an ANOVA followed by the Student-Newman-Keuls tests. For this purpose we used the software SigmaStat version 3.5.

## 3. Results

The mean weight of mice along the study is shown in Table 1. Control mice and those treated solely with BJ had a slight increase at the end of the study, while a 5.49 g decrease was noted in the AOM treated group at the third week of the assay, with a weight recovery in the last week. In the groups treated with BJ plus the carcinogen we observed a certain weight reduction which may be related to the toxicity of AOM, although cooperation between both chemicals cannot be discarded in light of the fact that the high reduction was observed with the high dose of BJ.

**Figure 1:**
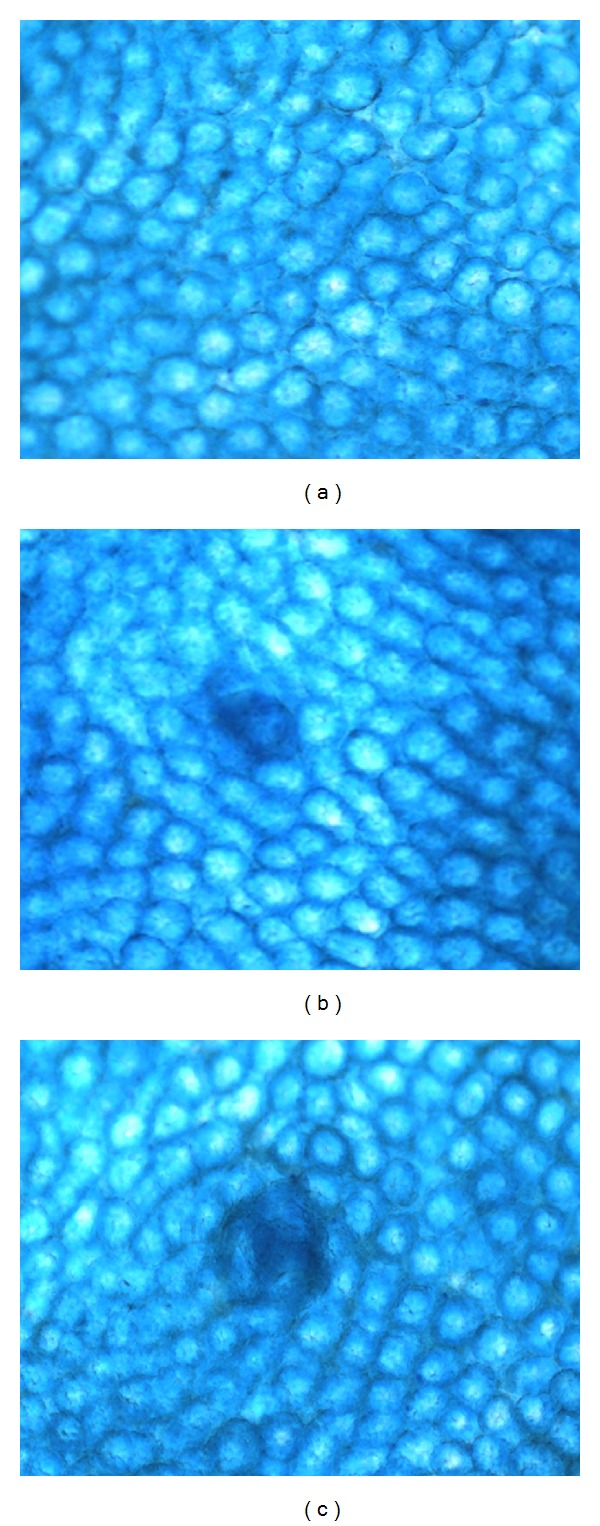
Aberrant crypts observed in the colon of mice treated with blueberry juice and azoxymethane. (a) Colon with normal crypts. (b) Colon with one aberrant crypt. (c) Colon with crypt foci formed by three aberrant crypts.

Table 2 shows the results obtained concerning the number of AC. Besides, Figure 1 shows various types of the observed crypts. We found no crypts in the control group, and we found a mean of 1.9 crypts in animals administered the high dose of BJ without statistical difference with respect to the previous group; these results are contrary to the 73.2 AC observed in mice administered AOM. On the other hand, animals administered the low dose of the juice plus AOM had a certain increase in the number of AC; however, in this group we also found a reduction of 64.7% in the number of AC with respect to the value obtained by administering the carcinogen. In the group treated with the intermediate dose of BJ plus AOM we observed a mean induction of 9.8 AC as well as an inhibition of 86.5% in comparison with the number observed in the treatment with AOM. Finally, mice treated with the high dose of BJ in addition to the carcinogen originated an unexpected increase of AC with no protective activity. On the contrary, the mean number in this group was more than 300 AC, which represents more than four times the number detected in the AOM treated group. The same table also shows the distribution of AC found in our study. Seven was observed to be the highest number of registered crypts. This number was found only in the AOM treated group. The administration of BJ alone gave rise to single or double crypts only, while the combination of BJ and AOM induced up to six crypts.

**Figure 2:**
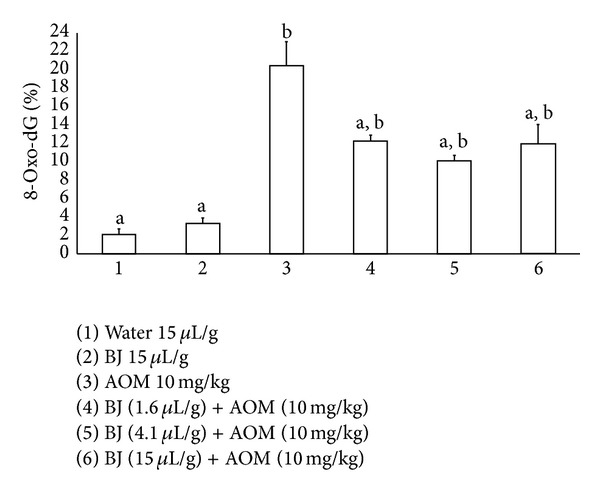
Quantification of 8-oxo-dG adducts in mice treated with blueberry juice (BJ) and azoxymethane (AOM). Each bar represents the mean ± SD obtained in the colon of each group. Eight mice per group. ^a^Statistically significant difference with respect to the AOM value; ^b^with respect to the control value. ANOVA and Student-Newman-Keuls tests, *P* ≤ 0.05.

The DNA immunohistochemical study made in the colon of the control and treated animals is shown in Figure 2. Mice of the control group and those administered BJ (15.0 *μ*L/g) were observed to have 2% and 3% nuclei positively marked for 8-oxo-dG, respectively, while animals treated with AOM gave rise to 21.2% 8-oxo-dG adducts. Besides, in the groups administered AOM plus BJ (1.6, 4.1, and 15.0 *μ*L/g) we determined a significant antioxidant effect, with a decrease of 40, 51, and 42% in the level of adducts with respect to the value obtained in the AOM treated group.

**Figure 3:**
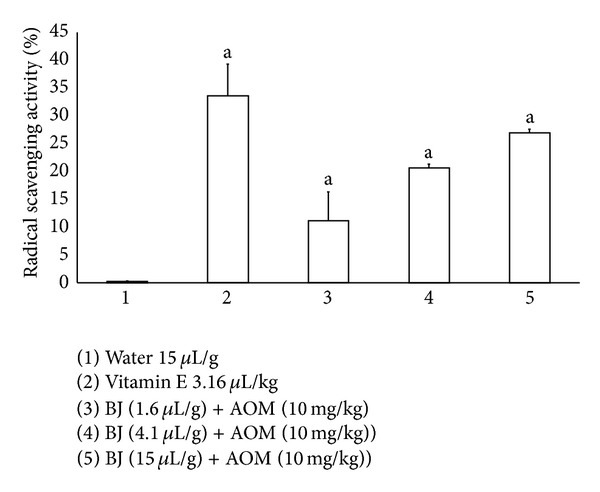
Results of* ex vivo* DPPH assay in mice treated with blueberry juice (BJ) and azoxymethane (AOM). Data were obtained in the serum of 8 animals per group. ^a^Statistically significant difference with respect to the control value. ANOVA and Student-Newman-Keuls tests, *P* ≤ 0.05.

Figure 3 shows the results obtained in the DPPH assay. We found a 33% counteraction produced by vitamin E in the presence of the DPPH radical, while the three tested doses of BJ (1.6, 4.1, and 15.0 *μ*L/g) gave rise to a dose-dependent decrease which corresponded to 11.1, 20.6, and 26.9%, respectively, in regard to the value obtained in the negative control.

## 4. Discussion

In the present study we evaluated various parameters to determine the toxic effect of AOM in mice as well as the level of protection exerted by BJ. In regard to animal weight, its decrease is generally accepted to suggest a systemic deterioration. This effect was observed in animals administered AOM only, a result that has also been reported by other authors, although such decrease has not always been found [21, 22]. BJ alone produced a slight increase in weight along the assay; however, when combined with AOM, the tested doses of BJ showed no protection on the strong toxicity induced by AOM. In the evaluation of other agents (including the blueberry constituent, epicatechin gallate), a similar type of result has been related to a deleterious effect of the carcinogen to the colon epithelial cells which is insufficiently prevented by the anticarcinogen and which provokes a decrease in the appetite of the experimental animals; this effect, however, has not been necessarily connected with the capacity of agents to prevent the induction of crypts [21–25].

Concerning the amount of AC observed in our study, we found that the administration of 15 *μ*L/g of BJ induced a low number of crypts which were in the range determined for the control group. This result clearly stated the innocuous action of the tested juice for inducing colon preneoplastic changes and was concordant with a number of reports that have evaluated different types of berries administered to animals as lyophilized products or in other forms, as well as with the negative results observed with constituents of blueberry, such as epicatechin [22, 26]. The described results contrast with the elevated number of AC as a consequence of administering AOM, a finding which is in line with the potent colon carcinogen capacity already described for this compound. Concerning the effect of the two low doses of BJ (1.6 and 4.1 *μ*L/g) administered to animals treated with AOM, we detected a significant prevention of the damage induced by the carcinogen. Such beneficial effect of blueberry has been determined before in rats, as well as with the use of constituents of the plant, including anthocyanins and pterostilbene [15, 27, 28]. Besides, the blueberry anticancer potential has also been determined in a model of rats given cyclic administrations of dextran sulphate sodium, a nongenotoxic compound that induces colitis and colitis associated neoplasia [29].

In our study we also determined a moderate but significant antioxidant capacity by the tested juice with the DPPH assay, as well as significant prevention of DNA oxidation, suggesting that this property of BJ may be involved in the reduction of genetic damage induced by AOM, which in subsequent steps may give rise to preneoplastic damage. The antioxidant capacity of blueberry has been attributed to the action of chemicals such as anthocyanins, catechins, quercetin derivatives, and benzoic and cinnamic acids [30]. However, this property is only one of the various parameters to be considered in the preventive process, as no change in the level of DNA oxidative adducts has been determined while other types of carcinogenic markers showed clear modifications [15]. This last information is in line with the fact that a number of molecular changes should be produced to state the neoplastic transformation, including alterations in genes involved in inflammation, proliferation, and apoptosis [31].

The inhibitory effect produced with the low doses of BJ is highly in contrast with the damage induced when the high dose of the juice was combined with AOM. Such preneoplastic potentiating effect deserves the mention of antecedents on the matter to put it into context. In genotoxicity, this type of enhancement has been described before, as well as its relationship with the used dose; such is the case of verapamil, a calcium antagonist agent that significantly increased the clastogenic effect of cyclophosphamide, acrylamide, and dioxidine in mouse; ascorbic acid also increased the number of micronuclei and sister chromatid exchanges induced by mitomycin C in human lymphocytes [32, 33]. Moreover, Dutra et al. [34] reported an enhancement of the genotoxic potential of doxorubicin in* Drosophila* by a cotreatment with tomato extract (*Lycopersicon esculentum*). Besides, polyphenols and tannins contained in areca nut have been suggested to participate in the ROS induced genotoxic, preneoplastic, and neoplastic effects that occur during autoxidation of areca nut polyphenols in the betel quid chewer's saliva, a crucial event for the initiation and promotion of oral cancer [35].

At the level of colon preneoplastic or tumor damage, a few unexpected results have also been reported. Blueberry powder (obtained from* V. angustifolium*) failed to protect against AOM-induced AC in rats treated with a damaging diet; moreover, authors found that blueberry increased the number of crypts in the distal colon of female animals [36]. Likewise, the administration of wheat bran was shown to enhance the rat colon carcinogenesis induced with 1,2-dimethylhydrazine, probably due to its hyperproliferative effect [37]. In fact, experimental evidence has suggested that a fibre-depleted diet may have cocarcinogenic potential in colorectal cancer, while a high fibre diet may have a protective, anticarcinogenic effect [38].

In spite of the numerous reports showing antioxidation as chemopreventive activity for berries and several of their constituents, we must recall that these chemicals may also have opposite effects; for example, flavonoids, such as apigenin and quercetin, as well as tannins are known to have DNA strand breaking abilities [39]. Resveratrol and ascorbic acid have also been reported to possibly cause DNA breakage through the generation of ROS [40, 41]. Heterogeneous effects of quercetin have also been reported; low doses were found to increase the production of micronuclei and chromosomal aberrations, contrary to the protective effect shown against mitomycin C genotoxicity with a high dose [42]. Moreover, the increase in the bioavailability of carcinogens may also modify the effect of, otherwise, protective agents [43].

In this complex panorama, the particular species of the plant and cultivar may play a role in the variability of the effect. Authors have found striking differences in the chemopreventive capacity among 13 types of berries as well as no correlation between their antiproliferative and antioxidant potential, differences that were even observed in plants of cultivars under the same environmental growing conditions [30, 44]. This variability was likewise observed among extracts with high proanthocyanidin content, which showed positive but also negative antiproliferative, antiradical, and protective effects against oxidative stress in three different cell lines [45, 46].

Our present results, together with those mentioned by other authors, point to a complex situation with respect to the BJ chemopreventive effect, in which the activity of specific polyphenols may have a role. Our report suggests the need to be cautious in regard to the use of BJ as well as to perform research about the possible comutagenic and cocarcinogenic effects between the juice and AOM, incorporating a number of factors, some of which have already been suggested in the above-mentioned reports, such as the influence of different doses, differences in strain sensibility, and the participation of the juice and its components in the molecular events involved in the precarcinogenesis process, as various genetic/epigenetic changes have a role in the transformation of a single epithelial cell within the crypt to the AC formation. Besides, other factors may participate in the damage, for example, the interaction of the evaluated agent with enzymes that catalyze the carcinogen-metabolic detoxification, the impairment of nucleotide excision repair mediated by failure of genes such as p53, or the influence of oxidative stress that may induce genomic instability and concur independently to generate the molecular damage required to induce neoplastic clones [47, 48].

In fact, BJ is a complex mixture in which synergistic or antagonistic interactions may occur and give different weight to the final effect. This has been suggested in regard to the content of anthocyanins, proanthocyanidins, and flavonol glycosides of cranberry, as well as with respect to phytochemicals from the quinine tree [49, 50]. Finally, our results also suggest a reflection on the boundaries of a chemopreventive event, a mutagenic/carcinogenic process, and a chemotherapeutic apoptosis-mediated event.

## Figures and Tables

**Table 1:** Weight (g) determined in mice treated with blueberry juice (BJ) and azoxymethane (AOM).

Week	Water 15 *μ*L/g	AOM 10 mg/kg	BJ 15 *μ*L/g	BJ + AOM 1.6 *μ*L/g + 10 mg/kg	BJ + AOM 4.1 *μ*L/g + 10 mg/kg	BJ + AOM 15 *μ*L/g + 10 mg/kg
0	30.25 ± 0.79	31.33 ± 0.67	29.72 ± 0.58	32.60 ± 0.75	32.35 ± 0.71	31.03 ± 0.69
1	30.45 ± 0.90	32.73 ± 0.97	29.23 ± 0.68	31.86 ± 0.73	31.83 ± 0.74	30.35 ± 0.68
2	30.79 ± 0.65	27.72 ± 1.15^a^	30.29 ± 0.89	30.22 ± 0.77	29.55 ± 1.91	25.85 ± 1.07^a^
3	32.15 ± 0.62	25.84 ± 1.23^a^	30.95 ± 1.01	28.78 ± 0.62^a,b^	27.46 ± 0.68^a^	24.16 ± 0.95^a^
4	33.81 ± 0.62	30.18 ± 1.11^a^	31.34 ± 1.22	31.24 ± 1.03	29.81 ± 0.79^a^	28.16 ± 0.95^a,b^

Values represent the mean ± SD obtained in 8 mice per group. The letters show significant statistical differences as follows: ^a^with respect to the control value (water) in the same week, ^b^with respect to value in the group treated with AOM in the same week. ANOVA and Student-Newman-Keuls tests, *P* ≤ 0.05.

**Table 2:** Aberrant crypts in mice treated with blueberry juice (BJ) and azoxymethane (AOM).

Group	Number of aberrant crypts							Mean ± SD
	1	2	3	4	5	6	7	
Water 15 *μ*L/g	0	0	0	0	0	0	0	0

AOM 10 mg/kg	57.14 ± 6.85	10 ± 1.44	3.28 ± 0.77	1.85 ± 0.41	0.14 ± 0.14	0.42 ± 0.42	0.42 ± 0.20	73.25 ± 1.44^a^

BJ 15 *μ*L/g	1.71 ± 0.89	0.28 ± 0.18	0	0	0	0	0	1.99 ± 0.54

BJ + AOM 1.6 *μ*L/g + 10 mg/kg	23.17 ± 3.37	1.83 ± 0.71	0.66 ± 0.49	0.16 ± 0.17	0	0	0	25.82 ± 1.19^a,b^

BJ + AOM 4.1 *μ*L/g + 10 mg/kg	8.5 ± 1.65	0.75 ± 0.37	0.37 ± 0.26	0.12 ± 0.13	0	0.12 ± 0.13	0	9.86 ± 0.51^a,b^

BJ + AOM 15 *μ*L/g + 10 mg/kg	299.5 ± 43.14	12 ± 2.72	4.16 ± 1.09	1 ± 0.26	0.33 ± 0.21	0	0	316.99 ± 9.48^a,b^

Values represent the mean ± SD obtained in the colon of 8 mice per group. ^a^Statistically significant with respect to the mean control value and ^b^with respect to the value of the AOM treated group. ANOVA and Student-Newman-Keuls tests, *P* ≤ 0.05.
